# Long Bone Mineral Loss, Bone Microstructural Changes and Oxidative Stress After *Eimeria* Challenge in Broilers

**DOI:** 10.3389/fphys.2022.945740

**Published:** 2022-07-18

**Authors:** Y. H. Tompkins, P. Teng, R. Pazdro, W. K. Kim

**Affiliations:** ^1^ Department of Poultry Science, University of Georgia, Athens, GA, United States; ^2^ Department of Foods and Nutrition, University of Georgia, Athens, GA, United States

**Keywords:** *eimeria*, bone health, bone mineral loss, bone quality, oxidative stress, broiler bone

## Abstract

The objective of this study was to evaluate the impact of coccidiosis on bone quality and antioxidant status in the liver and bone marrow of broiler chickens. A total of 360 13-day old male broilers (Cobb 500) were randomly assigned to different groups (negative control, low, medium-low, medium-high, and highest dose groups) and orally gavaged with different concentrations of *Eimeria* oocysts solution. Broiler tibia and tibia bone marrow were collected at 6 days post-infection (6 dpi) for bone 3-D structural analyses and the gene expression related to osteogenesis, oxidative stress, and adipogenesis using micro-computed tomography (micro-CT) and real-time qPCR analysis, respectively. Metaphyseal bone mineral density and content were reduced in response to the increase of *Eimeria* challenge dose, and poor trabecular bone traits were observed in the high inoculation group. However, there were no significant structural changes in metaphyseal cortical bone. Medium-high *Eimeria* challenge dose significantly increased level of peroxisome proliferator-activated receptor gamma (*PPARG*, *p* < 0.05) and decreased levels of bone gamma-carboxyglutamate protein coding gene (*BGLAP, p* < 0.05) and fatty acid synthase coding gene (*FASN*, *p* < 0.05) in bone marrow. An increased mRNA level of superoxide dismutase type 1 (*SOD1, p* < 0.05) and heme oxygenase 1 (*HMOX1, p* < 0.05), and increased enzyme activity of superoxide dismutase (SOD, *p* < 0.05) were found in bone marrow of *Eimeria* challenged groups compared with that of non-infected control. Similarly, enzyme activity of SOD and the mRNA level of *SOD1*, *HMOX1* and aflatoxin aldehyde reductase (*AKE7A2*) were increased in the liver of infected broilers (*p* < 0.05), whereas glutathione (GSH) content was lower in the medium-high challenge group (*p* < 0.05) compared with non-challenged control. Moreover, the mRNA expression of catalase (*CAT*) and nuclear factor kappa B1 (*NFKB1*) showed dose-depend response in the liver, where expression of *CAT* and *NFKB1* was upregulated in the low challenge group but decreased with the higher *Eimeria* challenge dosage (*p* < 0.05). In conclusion, high challenge dose of *Eimeria* infection negatively affected the long bone development. The structural changes of tibia and decreased mineral content were mainly located at the trabecular bone of metaphyseal area. The change of redox and impaired antioxidant status following the *Eimeria* infection were observed in the liver and bone marrow of broilers.

## Introduction

Avian coccidiosis is one of the top prevalent enteric diseases in the poultry industry. Especially in the modern broiler production, the high-density, small confinement, warm, and humid animal housing accelerate the dispersal, transmission, and outbreak of coccidiosis, making this issue hard to eradicate (Chapman 2014; Blake et al., 2020). Coccidiosis is a parasite disease caused by parasites of the genus *Eimeria* that can cause intestinal damage leading to inflammation and nutrient malabsorption (Gautier et al., 2019). The infection with *Eimeria* spp. results in growth retardation and mortality, which creates 13 billion dollars in losses by its detriment to production and increases the cost (Amerah and Ravindran, 2015). The prevention and control of coccidiosis outbreaks are not only achieved by careful management practices, but also the use of in-feed anticoccidial drugs or vaccines, alone or in combination. However, because of the market demand, the use of antibiotic-free diets leads to numerous challenges, such as control and treatment of enteric and systemic diseases (Dalloul and Lillehoj, 2006; Blake and Tomley, 2014; Cervantes, 2015). Other than anorexia or nutrient malabsorption, the pathogenicity of coccidiosis is also associated with the response from immune system which generates reactive oxygen species (ROS) in chicken (Georgieva et al., 2006; Georgieva et al., 2011; Gautier et al., 2019). The parasite infection causes an imbalance between endogenous antioxidant defense and free radical production, which leads to depletion of antioxidant enzymes and reduction of glutathione (GSH) level (Surai, 2019). The unbalanced status results in increased lipid peroxidation and DNA damage which can cause apoptosis of intestinal cells and affect the health status and productivity of poultry (Estevez, 2015; Mishra and Jha, 2019). The first lines of antioxidant defense system including catalase (CAT), superoxide dismutase (SOD), and glutathione peroxidase (GPX) are indispensable for protecting the body from the damage caused by free radicals, especially superoxide anion radicals, during *Eimeria* infection (Georgieva et al., 2006). Dietary manipulations, such as optimizing amino acids profile or adding dietary supplements are potential strategies to support broilers against coccidiosis induced oxidative stress (Arczewska-Wlosek et al., 2018; Gautier et al., 2019). In broilers, nutrient supplements such as vitamins, antioxidants, and trace minerals can alleviate the negative effect caused by oxidative stress (Arczewska-Wlosek et al., 2018; Santos et al., 2020).

Meanwhile, the incidence of physical abnormalities in bone of broilers has been noted. Because the fast-growing broilers are characterized by poor calcification and high porosity of long bone, severe duodenum and upper jejunum damage caused by *Eimeria* infection intensifies the bone health issues in the modern poultry industry (Sakkas et al., 2018; Oikeh et al., 2019). Bone mineral loss caused by *Eimeria* spp. in broilers has been previously linked to nutrition malabsorption, especially the reduced absorption of calcium, phosphate, and several important trace minerals for optimal bone growth (Turk and Stephens, 1967; Turk and Stephens, 1969; Turk and Stephens, 1970; Turk, 1973; Joyner et al., 1975). Recent studies have revealed a more profound understanding in regards to bone loss after *Eimeria* infection, where suppressed fat absorption resulted in depressed levels of fat-soluble vitamins; thus, an increase in bone resorption level was detected (Akbari Moghaddam Kakhki et al., 2019; Sakkas et al., 2019). Studies in human and mice also indicated that oxidative stress response caused by gastrointestinal infection could lead to inhibition of mineralization and osteogenesis, and activation of bone resorption, subsequently causing bone loss and structural changes (Basu et al., 2001; Domazetovic et al., 2017). Bone architectural organization is an independent marker that can precisely reflect bone turnover, however, how does the *Eimeria* infection change biomechanical properties on the specific bone region has not been documented extensively to date, and the etiology behind it is not fully understood (Brandi, 2009; Henkelmann et al., 2017). We reported that the increasing infection severity of *Eimeria* spp. linearly reduced nutrient digestibility and body weight of birds. The increased gut permeability and lesion scores in response to the graded levels of *Eimeria* infection were also found and presented in our recent publication (Teng et al., 2020). The objective of this study was to further evaluate the negative impact of coccidiosis on bone traits in broiler chickens. A new approach, micro-CT scanning and analyses, was taken in assessing the three-dimensional structure to provide in-depth and comprehensive understanding the pathogenetic mechanisms of bone disorders with acute intestinal pathogen infections in avian species.

## Materials and Methods

### Ethics Statement

All experiments followed the guidelines of the Institutional Animal Care and Use Committee and was conducted at the Poultry Research Farm, University of Georgia, Athens, GA. The protocol was approved by the Institutional Animal Care and Use Committee at the University of Georgia.

### Experimental Design

Management and diet formulation as previously described (Teng et al., 2020). Briefly, a total of 360 male broiler chicks were randomly allocated to five treatments with six replicates and twelve birds per cage. The birds were gavaged with 1 ml of water for a control and 1 ml of different concentrations of *Eimeria* solutions for challenge groups at 13 days of age. Mixed *Eimeria* spp. oocyst solutions were pre-prepared for the Low group as the lowest challenge dose with 6,250 oocysts of *E. maxima*, 6,250 oocysts of *E. tenella* and 31,250 oocysts of *E. acervulina*; the Med-low group as the medium-low challenge dose with 12,500 oocysts of *E. maxima*, 12,500 oocysts of *E. tenella* and 62,500 oocysts of *E. acervulina*; the Med-high group as the medium-high challenge dose with 25,000 oocysts of *E. maxima*, 25,000 oocysts of *E. tenella* and 125,000 oocysts of *E. acervulina*; and the High group as the highest challenge dose with 50,000 oocysts of *E. maxima*, 50,000 oocysts of *E. tenella*, and 250,000 oocysts of *E. acervulina* (Table 1). All chicks were raised under the same environmental conditions according to the Cobb 500 broiler management guide (Cobb, 2019). All chicks were fed the same basal diet and allowed to consume feed and water on an *ad libitum* basis. Starter (0–12 days of age) and grower (13–19 days of age) diets were formulated to meet Cobb 500 nutrient requirements as previously described (Teng et al., 2020). A total of 30 birds (1 bird per replicate cage) were selected and euthanized by cervical dislocation at six dpi (19 days of age), and tibia bone and liver samples were collected and snap-frozen in liquid nitrogen and kept in −80°C until processing.

**TABLE 1:** *Eimeria* spp. challenge dosage (Unit: oocysts/chick).

Treatment group^a^	*E. maxima*	*E. tenella*	*E. acervulina*	Total Concentration	Challenge Dosage
Control	0	0	0	0	Non-challenge
Low	6,250	6,250	31,250	43,750	Lowest challenge dose
Med-low	12,500	12,500	62,500	87,500	Medium-low challenge dose
Med-high	25,000	25,000	125,000	175,000	Medium-high challenge dose
High	50,000	50,000	250,000	350,000	Highest challenge dose

a Low, the lowest challenge dose; Med-low, the medium-low challenge dose; Med-high, the medium-high challenge dose; High, the highest challenge dose.

### Antioxidant Study by Enzyme-Linked Immunosorbent Assay

SOD and CAT enzyme activities in the liver and bone marrow of 30 samples (6 samples per treatment group) were analyzed using superoxide dismutase assay and catalase assay kits (Cayman chemical, Superoxide dismutase assay kit, item No. 706002, Catalase Assay Kit, item No. 707002, AnnArbor, MI, United States), following the manufacturer’s instructions. Approximately 100 mg of each sample was homogenized in 1 ml of cold sample buffer (20 mM HEPES buffer, pH 7.2, 2 mM EGTA, 10 mM mannitol, and 70 mM sucrose). The homogenized sample was centrifuged at 1,500 × g for 5 minutes at 4°C, and the supernatant was collected for analyses. All supernatant samples were diluted by using the sample buffer before the ELISA assays. Samples were measured by spectrophotometer (SpectraMax ABS Plus, Softmax Pro seven software, Molecular devices, San Jose, CA) at wavelength of 450 nm for SOD activity assay, and at 540 nm for CAT assay. For protein quantification assay (Pierce™ BCA Protein Assay Kit, Ref. 23,227, Thermo Scientific, Rockford, IL, United States), the supernatants were diluted before the assay, and Bovine Serum Albumin (2 mg/ml) was used as the protein standard, and enzyme activity was normalized to the total protein content for the final calculation. The protein samples were diluted and placed in duplicate and read in a spectrophotometer (SpectraMax, San Jose, CA) at wavelength of 562 nm.

### High-Performance Liquid Chromatography

High-performance liquid chromatography (HPLC) setting and reading for measuring antioxidative parameters were as previously described (Gould et al., 2018). Immediately after collecting liver and tibia marrow samples, all samples were snap-frozen in liquid nitrogen. Within 24 h, all harvested tissues were homogenized in PBS containing 10 mM diethylenetriaminepentaacetic acid (DTPA) and promptly acidified as previously described (Park et al., 2010). Samples were stored at -80 °C for HPLC analyses. Briefly, glutathione (GSH) and glutathione disulfide (GSSG) were quantified in each sample by HPLC coupled with electrochemical detection (Dionex Ultimate 3,000, Thermo Scientific, Waltham, MA, United States). The cell was set at + 1,600 mV with a cleaning potential of +1900 mV between the samples. The mobile phase consisted of 4.0% acetonitrile, 0.1% pentafluoropropionic acid, and 0.02% ammonium hydroxide. The flow rate was maintained at 0.5 ml/min, and injection volumes were set at 2.0 µl for bone marrow samples. Peaks were quantified using external GSH and GSSG standards and the Chromeleon Chromatography Data System Software (Dionex Version 7.2, Thermo Scientific, Germering, Germany). Total glutathione was determined by calculating GSH + 2GSSG, and levels of total glutathione, GSH, and GSSG were all standardized to total protein content (Pierce™ BCA Protein Assay Kit). The protein samples were diluted and placed in duplicate and read in a spectrophotometer (SpectraMax, San Jose, CA) at wavelength of 562 nm.

### Micro-Computed Tomography

To evaluate bone morphologic changes in the broiler, 30 samples (6 samples per treatment group) were randomly chosen for micro-Computed Tomography (micro-CT) microarchitectural scanning. The right tibias were scanned according to a standard protocol at 80 kV and128 µA, and a 0.5 mm aluminum filter, and analyses were performed with a SkyScan 1,172 (SkyScan, Kontich, Belgium). The scanned images were captured with a 360° complete rotation and an 18 min of acquisition time at 26 µm pixel size. 2-D images were transferred to CTAn software (CTAn, SkyScan, Aartselaar, Belgium) for structure construction and quantification as previously described (Chen and Kim, 2020). Trabecular and cortical bones of the metaphysis were analyzed. The analysis parameters are listed in Table 2. All images were post-operated to isolate trabecular bone from cortical bone and preserve its morphology using a threshold of 800 manually. Average bone mineral content (BMC), bone mineral density (BMD), and bone micro-architectural parameters of each treatment group were taken from the same region of interest (ROI). The whole bone length and bone diaphysis width were measured by using CTAn ruler tool which measures straight line distance. Controlling the location, four measurements were conducted on each sample by using CTAn ruler tool, the mean thickness of cortical bone was used for statistical analysis.

**TABLE 2:** Definition and description of bone microstructure by using micro-CT method (Bouxsein et al., 2010).

Abbreviation	Variable	Unit	Description
BMC	Bone mineral content	g	The amount of the solid objects (bone minerals that mostly included calcium and phosphorous) within the region of interest
BMD	Bone mineral density	g/mm^2^	The ratio of bone minerals within a mixed bone-soft tissue region
TV	Total volume	mm^3^	Volume of the entire region of interest
BV	Bone volume	mm^3^	Volume of the region segmented as bone
BS	Bone surface	mm^2^	Surface area of all solid objects (bone tissue) within the total tissue volume
BV/TV	Bone volume fraction	%	Ratio of the solid objects (bone tissue) volume to the total volume of the region of interest
BS/BV	Specific bone surface	mm^2^/mm^3^	Ratio of the segmented bone surface to the mineralized bone volume
BS/TV	Bone surface density	%	Ratio of the segmented bone surface to the total volume of the region of interest
Tb. N	Trabecular number	1/mm	Measure of the average number of trabeculae per unit length
Tb. Th	Trabecular thickness	mm	Mean thickness of trabeculae osseous structure. It assessed using direct 3D methods
Tb. Sp	Trabecular spacing	mm	Mean space between trabeculae (marrow space), assessed using direct 3D methods
SMI	Structure model index	-	An indicator of the structure of trabeculae; Parallel plates was defined as 0 and cylindrical rods was rated as 3
Tb.pf	Trabecular pattern factor	1/mm	Describes quantitatively trabecular connectivity
Conn. Dn	Connectivity density	1/mm^3^	A measure of the degree of connectivity of trabeculae normalized by TV
Po (op)	Cortical porosity (open pore)	%	In a given cortical region, the volume of open pores (Po.V, mm^3^) ÷ total volume of cortical bone compartment (Ct.V, mm^3^)
Po.V (op)	Open pore volume	mm^3^	The volume of the open pores
Po.V (tot)	Total pore volume	mm^3^	The volume of all pores
Po (tot)	Total cortical porosity	%	In a given cortical region, the volume of pores (Po.V, mm^3^) ÷ total volume of cortical bone compartment (Ct.V, mm^3^)

### Real-Time qPCR Analysis for Gene Expression in the Bone Marrow

Right tibia bones were opened, and bone marrow samples were collected and stored at −80°C until RNA isolation (n = 6). Bone marrow and liver total RNA were extracted by using Qiazol reagents (Quiagen, Germantown, MD, United States) according to the manufacturer’s instructions. Nano-Drop 1,000 Spectrophotometer (ThermoFisher Scientific, Pittsburgh, PA, United States) was used to determine the quantity of extracted RNA. The cDNA was synthesized from total RNA (2000 ng) using high-capacity cDNA reverse transcription kits (Thermo Fisher Scientific, Waltham, MA, United States). Real-time reverse transcription polymerase chain reaction (Real-time RT-PCR) was used to measure mRNA expression. Primers were designed using the Primer-BLAST program (https://www.ncbi.nlm.nih.gov/tools/primer-blast/). The specificity of primers was validated by PCR product sequencing and previously published (Table 3). Primer quality was verified through melting curve analysis and gel electrophoresis in this study. Real-time qPCR was performed on an Applied Biosystems StepOnePlus™ (Thermo Fisher Scientific, Waltham, MA, United States) with iTaq™ universal SYBR Green Supermix (BioRad, Hercules, CA, United States) using the following conditions for all genes: 95°C for 10 min followed 40 cycles at 95°C for 15 s, annealing temperature (Table 3) for 20 s, and extending at 72°C for 1 minute.

**TABLE 3:** Nucleotide sequences of the primers used for real-time qPCR.

Gene^a^	Primer Sequence (5′-3′)	Product length (bp)	Annealing temperature (°C)	Accession #	Gene references
GAPDH	F-GCTAAGGCTGTGGGGAAAGT R-TCAGCAGCAGCCTTCACTAC	161	55	NM_204,305.1	Su et al. (2020)
ACTB	F-CAACACAGTGCTGTCTGGTGGTA R-ATCGTACTCCTGCTTGCTGATCC	205	61	NM_205,518.1	Teng et al. (2020)
NFKB1	F-GAAGGAATCGTACCGGGAACA R-CTCAGAGGGCCTTGTGACAGTAA	131	59	XM_015,285,418.2	Su et al. (2020)
RUNX2	F-ACTTTGACAATAACTGTCCT R-GACCCCTACTCTCATACTGG	192	60	XM_015,285,081.2	Adhikari et al. (2020)
PPARG	F-GAGCCCAAGTTTGAGTTTGC R-TCTTCAATGGGCTTCACATTT	131	58	XM_025,154,400.1	Chen et al. (2021)
FASN	F-AGAGGCTTTGAAGCTCGGAC R-GGTGCCTGAATACTTGGGCT	127	60	NM_205,155.3	Su et al. (2020)
FABP4	F-GCAGAAGTGGGATGGCAAAG R- GTT​CGC​CTT​CGG​ATC​AGT​CC	153	60	NM_204,290.1	Chen et al. (2021)
BGLAP	F-GGATGCTCGCAGTGCTAAAG R-CTCACACACCTCTCGTTGGG	142	57	NM_205,387.3	Adhikari et al. (2020)
SOD1	F-ATTACCGGCTTGTCTGATGG R-CCTCCCTTTGCAGTCACATT	173	58	NM_205,064.1	Oh et al. (2019)
CAT	F-ACTGCAAGGCGAAAGTGTTT R-GGCTATGGATGAAGGATGGA	222	60	NM_001,031,215.1	Oh et al. (2019)
AKR7A2	F-CAAACTGCAGGGTTCTCTTG R-GAAGTAGTTGGGGCAGTCGT	234	60	NM_205,344.1	Lee et al. (2018)
HMOX1	F-CTGGAGAAGGGTTGGCTTTCT R-GAAGCTCTGCCTTTGGCTGTA	166	60	XM_417,628.2	Oh et al. (2019)
GPX1	F-AACCAATTCGGGCACCAG R-CCGTTCACCTCGCACTTCTC	122	60	NM_001,277,853.2	Oh et al. (2019)

a GAPDH: glyceraldehyde-3-phosphate dehydrogenase; ACTB: actin beta; PPARG: peroxisome proliferator-activated receptor gamma; FASN: fatty acid synthase; SREBP1: sterol regulatory element-binding transcription factor 1; BGLAP: bone gamma-carboxyglutamate protein; RUNX2: runt-related transcription factor 2; FABP4: adipose tissue fatty acid binding protein four; NFKB1: nuclear factor kappa B subunit one; CAT: catalase; SOD1: superoxide dismutase type 1; GPX1: glutathione peroxidase one; HMOX1: heme oxygenase one; and AKR7A2: aflatoxin aldehyde reductase.

The geomeantric means of glyceraldehyde-3-phosphate dehydrogenase (*GAPDH*) and actin beta (*ACTB*) were used as housekeeping genes for normalization, and the stability of the housekeeping genes was confirmed by their consistent Ct values among the treatments (*p* > 0.1) (Vandesompele et al., 2002). Details of primer sequences used for the experiment are presented in Table 3. Peroxisome proliferator-activated receptor gamma (*PPARG*), fatty acid synthase (*FASN*), adipose tissue fatty acid binding protein 4 (*FABP4*) and sterol regulatory element-binding transcription factor 1 (*SREBP1*) were used as early markers of adipogenic differentiation and fatty acid synthesis, and bone gamma-carboxyglutamate protein (*BGLAP*) and runt-related transcription factor 2 (*RUNX2*) were used as osteogenic marker genes in the bone marrow. Nuclear factor kappa B subunit 1 (*NFKB1*) and antioxidant enzyme protein coding genes including catalase (*CAT*), superoxide dismutase type 1(*SOD1*), glutathione peroxidase 1 (*GPX1*), heme oxygenase 1 (*HMOX1*), and aflatoxin aldehyde reductase (*AKR7A2*) were used to determine the antioxidant enzyme activity and oxidative stress status (Lee et al., 2018). Samples were run in triplicate, and relative gene expression data were analyzed using the 2^−ΔΔCt^ (Livak et al., 2001). The mean ΔCt of each marker gene from the control group was used to calculate the ΔΔCt value, and 2^−ΔΔCt^ expression levels were normalized to one for the control group, and expression levels of the other treatment groups were presented as fold change relative to the control group.

### Statistical Analysis

All experimental data were expressed as mean with standard errors of the means (SEM). Data were tested for homogeneity of variances and normality of studentized residuals. The differences between the treatment groups were analyzed by one-way ANOVA, and the means were analyzed statistically by Tukey’s test using JMP Pro14 (SAS Institute, Cary, NC, United States). A *p ≤* 0.05 was considered statistically significant, and 0.05 ≤ *p* ≤ 0.1 were also presented to show the trending toward statistical significance (Thiese et al., 2016; Serdar et al., 2021). To evaluate the effects of increasing oocysts inoculation doses on responses of each parameter, the linear and quadratic regression were analyzed using an ordered logistic regression model with inoculated number of oocytes as a fixed factor and broiler per pen as the experimental unit. The comparisons between non-challenge control and pooled challenged groups (Low, Med-low, Med-high, and High) were calculated by unpaired *t*-test with Welch’s correction. Pair wise correlations (JMP Pro14) were evaluated for bone micro-CT and antioxidant variables. Statistical significance was set at *p ≤* 0.05.

## Results

### Bone Microstructural Changes in Response to Increasing Doses of *Eimeria* Oocysts

There were no statistically significant differences in the whole tibia length, tibia diaphysis width and the thickness of cortical bone among treatment groups at six dpi (Table 4). And all the micro-CT results are presented in Table 5. For the total bone structure of metaphysis, the lowest BMC (ANOVA, *p* = 0.025; linear regression, *p =* 0.012, *R* ^2^ = 0.205), BMD (ANOVA, *p* = 0.002; linear regression, *p <* 0.001, *R* ^2^ = 0.342), and the lowest bone volume fraction (BV/TV; ANOVA, *p =* 0.023) ratio were detected in the High group in response to increased challenge dose.

**TABLE 4:** Tibia length, width, and cortical bone thickness.

Unit (mm)	Bone length	Bone width	Cortical bone thickness^a^
Control	54.497	4.959	0.841
Low	54.436	4.969	0.850
Med-low	55.413	4.958	0.898
Med-High	55.109	4.969	0.841
High	55.092	5.086	0.873
SEM	0.244	0.051	0.021
ANOVA	0.226	0.925	0.836

a Cortical thickness: a mean thickness of cortical mid-shaft.

**TABLE 5:** Tibial metaphysis microstructure changes with increasing challenge dose of mixed *Eimeria* spp. oocysts.

	Items^a^ ^,^ ^b^	Unit	Control	Low	Med-low	Med-High	High	SEM	ANOVA
**Total**	BMC	G	61.244 ^ab^	70.906^a^	68.282 ^ab^	67.241 ^ab^	51.567^b^	2.139	0.025
	BMD	g/mm^2^	0.209^a^	0.209^a^	0.215^a^	0.213^a^	0.170^b^	0.004	0.002
	TV	mm^3^	295.216	339.192	318.777	312.631	303.064	6.177	0.284
	BV	mm^3^	89.331	100.332	108.552	101.309	85.588	3.196	0.134
	BS	mm^2^	1,260.402	1,419.550	1,415.952	1,333.981	1,116.761	48.366	0.250
	BV/TV	%	29.590 ^ab^	29.628 ^ab^	34.105^a^	32.069 ^ab^	28.161^b^	0.667	0.023
	BS/BV	mm^2^/mm^3^	14.018	14.114	13.047	13.281	13.025	0.224	0.360
	BS/TV	%	4.160	4.171	4.454	4.236	3.671	0.105	0.202
**Trabecular**	BMC	G	2.812^a^	2.646^a^	1.839^ab^	2.462^a^	1.087^b^	0.161	0.001
	BMD	g/mm^2^	0.087^a^	0.096^a^	0.068^ab^	0.083^a^	0.038^b^	0.005	<0.001
	TV	mm^3^	178.487	205.576	174.901	178.060	188.858	3.882	0.065
	BV	mm^3^	5.802	7.704	6.717	6.086	4.863	0.331	0.073
	BS	mm^2^	295.527^a^	385.188^ab^	340.596 ^ab^	321.624 ^ab^	258.360^b^	14.230	0.048
	BV/TV	%	3.238	3.710	3.863	3.424	2.59072	0.156	0.075
	BS/BV	mm^2^/mm^3^	52.046	50.578	50.831	52.900	54.140	0.643	0.394
	Tb. N	1/mm	0.389 ^ab^	0.435 ^ab^	0.464^a^	0.421 ^ab^	0.310^b^	0.017	0.025
	Tb. Th	Mm	0.082	0.085	0.083	0.081	0.083	0.001	0.846
	Tb. Sp	Mm	2.848	2.513	2.364	2.376	2.935	0.087	0.100
	SMI	-	2.622^b^	2.617^b^	2.577^b^	2.661 ^ab^	2.759^a^	0.017	0.002
	Tb.pf	1/mm	18.814 ^ab^	18.238 ^ab^	17.943^b^	19.635 ^ab^	21.084^a^	0.360	0.029
	Conn. Dn	1/mm^3^	10.158 ^ab^	11.076^a^	11.852 ^ab^	10.389 ^ab^	8.077^b^	0.407	0.035
**Cortical**	BMC	G	44.029	47.190	51.033	46.949	41.989	1.388	0.306
	BMD	g/mm^2^	0.412	0.401	0.404	0.402	0.409	0.876	0.876
	TV	mm^3^	108.061	118.168	126.627	117.158	103.270	3.955	0.386
	BV	mm^3^	79.177	87.435	93.899	87.243	78.179	2.614	0.298
	BV/TV	%	73.945	74.158	74.263	74.701	76.121	0.537	0.741
	Po (op)	%	25.951	25.736	25.604	25.171	23.763	0.536	0.737
	Po.V (op)	mm^3^	28.767	30.605	32.559	29.757	24.971	1.455	0.589
	Po.V (tot)	mm^3^	28.884	30.733	32.727	29.915	25.091	1.462	0.588
	Po (tot)	%	26.055	25.842	25.738	25.299	23.879	0.537	0.741

a Low, the lowest challenge dose; Med-low, the medium-low challenge^a^dose; Med-high, the medium-high challenge dose; High, the highest challenge dose.

b BMC, bone mineral content; BMD, bone mineral density; TV, total bone volume; BV, bone volume (TV, minus bone marrow volume); BS, bone surface area; BV/TV, bone volume/total volume; BS/BV, bone surface/total volume; BS/TV, bone surface/total volume; Tb. N, trabecular number; Tb. Th, trabecular bone thickness; Tb. sp, trabecular spacing; SMI, structural model index; Tb. Pf, trabecular pattern factor; Conn. dn, connectivity density; Po.V (tot), total volume of pore space; Po. V (op), volume of open pore; Po (tot), porosity rate (percent).

^a, ab,b^ Treatments with different letters means a significantly difference between treatments by using Tukey’s HSD, test, *p <* 0.05, N = 6.

**FIGURE 1:**
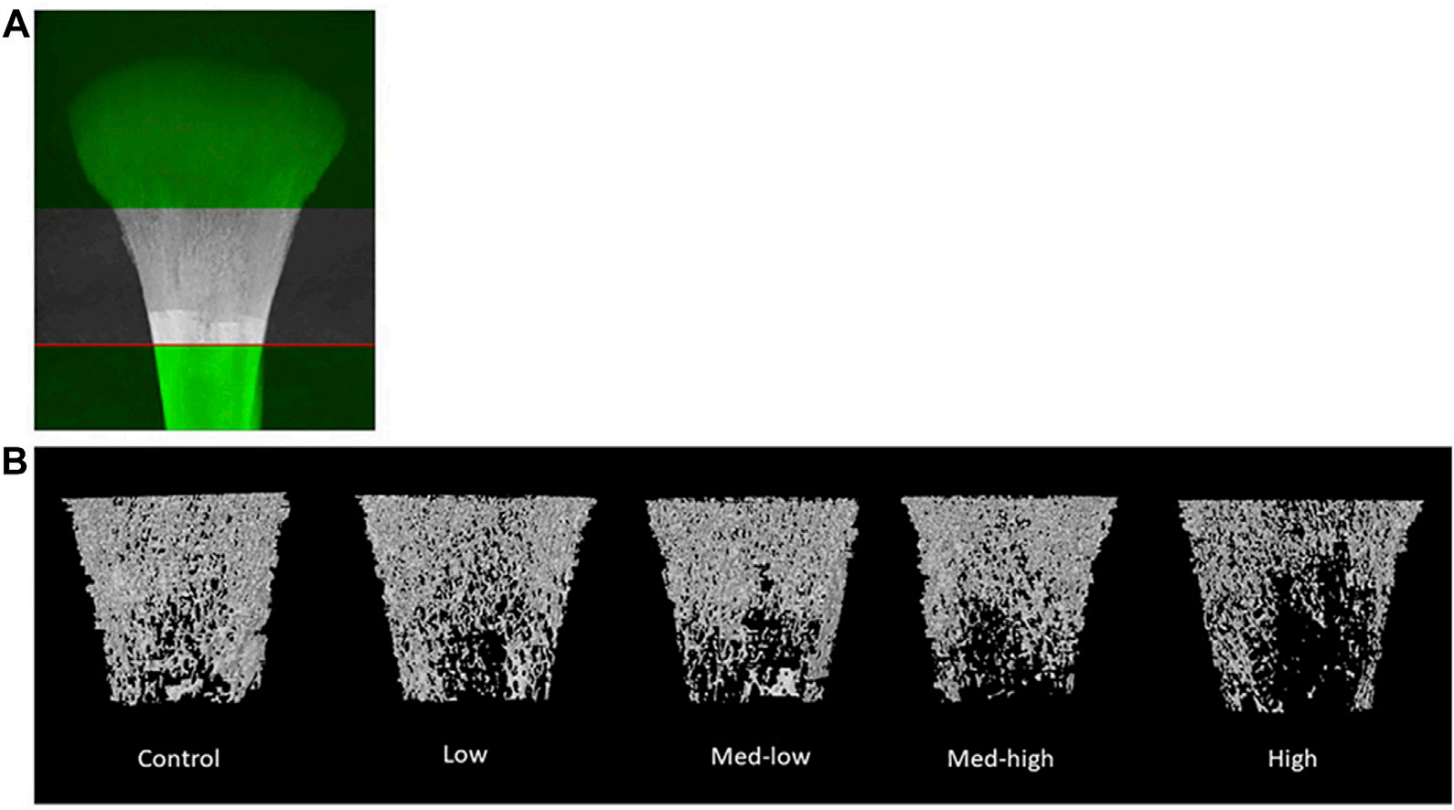
**(A)** The selection of metaphyseal region of interest. **(B)** Representative reconstructed 2D images of broiler tibia metaphysis at six dpi (19 days of age). Metaphyseal structure analysis showed a lower bone mineral content and density coupled with impaired trabecular bone traits following the higher inoculation doses of *Eimeria*. The negative impact of Eimeria infection on bone traits was mainly located at metaphyseal trabecular bone.

The microstructure changes in metaphysis were mainly attributed to impaired trabecular bone traits caused by higher *Eimeria* challenge dosage, including lower BMC (Figure 1; ANOVA, *p =* 0.001; linear regression, *p <* 0.001, *R* ^2^ = 0.362), lower BMD (ANOVA, *p* < 0.001; linear regression, *p* < 0.001, *R* ^2^ = 0.402), smaller bone surface (BS; ANOVA, *p =* 0.048), lower trabecular number (Tb. N; ANOVA, *p* = 0.025; linear regression, *p* = 0.021, *R* ^2^ = 0.177), lower connectivity density (Conn. Dn; ANOVA, *p* = 0.035; linear regression, *p* = 0.014, *R* ^2^ = 0.243), and higher rating structure model index (SMI; ANOVA, *p* = 0.002; linear regression, *p* < 0.001, *R* ^2^ = 0.387), higher rating of trabecular pattern factor (Tb. pf; ANOVA, *p* = 0.029; linear regression, *p* = 0.003, *R* ^2^ = 0.268). However, the microstructure of metaphyseal cortical bone was not significantly affected by *Eimeria* infection (ANOVA, *p* > 0.050).

### Gene Expression Changes of Bone Formation and Adipogenic Markers in the Bone Marrow

**FIGURE 2:**
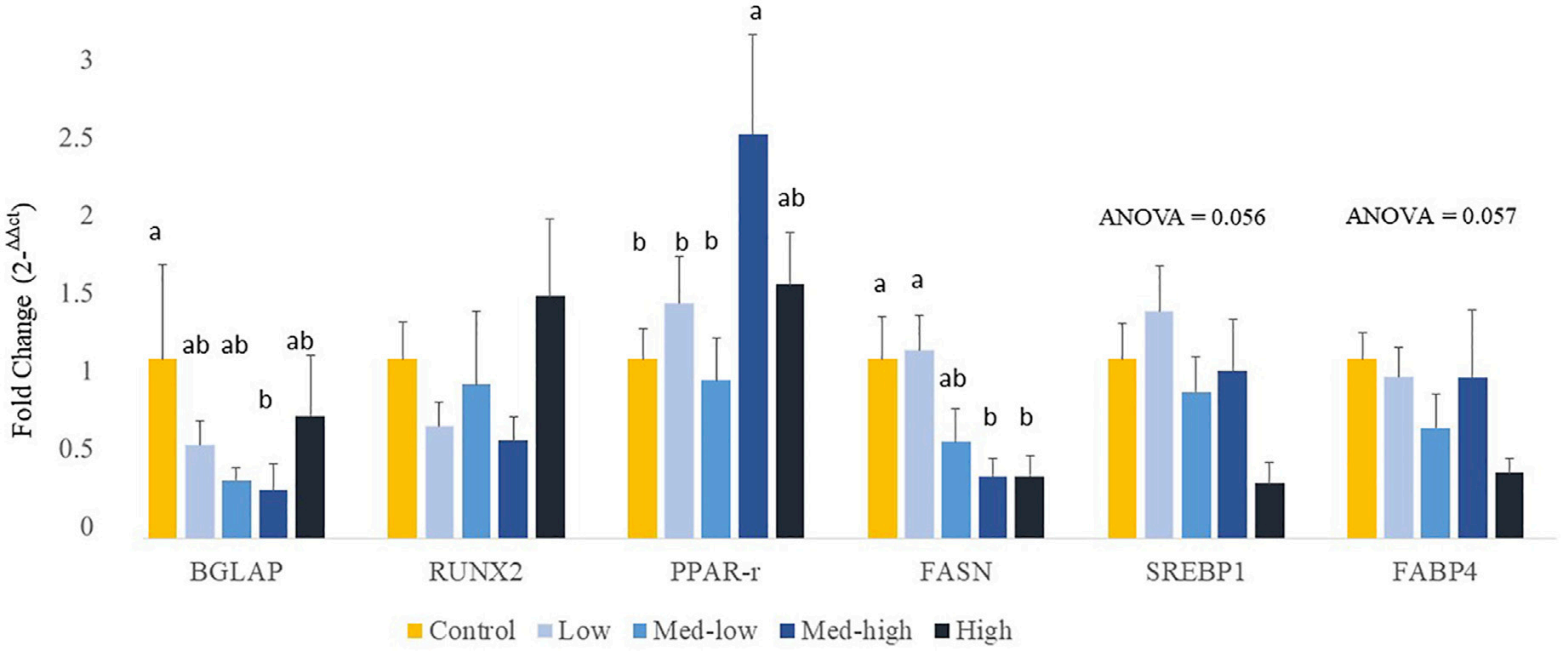
Effects of increasing oocysts dose of *Eimeria* mix on osteogenesis, adipogenesis and fatty acid synthesis gene expression in bone marrow of broilers. Low, the lowest challenge dose; Med-low, the medium-low challenge dose; Med-high, the medium-high challenge dose; High, the highest challenge dose. BGLAP, bone gamma-carboxyglutamate protein; RUNX2, runt-related transcription factor 2; PPARG, peroxisome proliferator-activated receptor gamma; FASN, fatty acid synthase; SREBP1: sterol regulatory element-binding transcription factor 1; FABP4: adipose tissue fatty acid binding protein 4. ^a, ab, b^ Treatments with different letters means a significantly difference between treatments by using Tukey’s HSD test, *p <* 0.05, N = 6.

The expression of protein coding genes that are involved in bone formation or adipocyte differentiation was measured (Figure 2). For bone growth gene markers, results showed a significant downregulation of *BGLAP* with increased inoculation levels (ANOVA*, p* = 0.020; linear regression, *p* = 0.029, *R* ^2^ = 0.396), where the lowest level of *BGLAP* was detected in the Med-High group. The mRNA expression of *RUNX2* was not affected by different doses of *Eimeria* oocysts challenge (ANOVA, *p* > 0.100). For adipogenic gene expression, the expression of *PPARG* was significantly increased by the Med-high dose of challenge when compared with the Control, the Low and the Med-low groups (Figure 2; ANOVA*, p* = 0.047). The expression of *SREBP1* (Figure 2; ANOVA*, p* = 0.056*;* linear regression*, p* = 0.008, *R* ^2^ = 0.226), *FABP4* (Figure 2; ANOVA*, p =* 0.057*;* linear regression*, p* = 0.004, *R* ^2^ = 0.279), and *FASN* (ANOVA*, p =* 0.013; linear regression*, p* = 0.005, *R* ^2^ = 0.261) were down-regulated in response to graded inoculation doses.

### Antioxidant Status in the Bone Marrow in Response to *Eimeria* Challenge

In the bone marrow, SOD enzyme activity increased in response to graded levels of oocysts challenge and showed the highest response to the Med-high challenge dose (Table 6; ANOVA, *p* = 0.027). However, the CAT enzyme activity was not significantly affected by the *Eimeria* challenge (Table 6; *p* > 0.050) in bone marrow. The bone marrow GSSG levels did not significantly changed by *Eimeria* infection, but it exhibited a negative response to increasing inoculation doses (Table 6; ANOVA, *p* > 0.050; linear regression, *p* = 0.039, *R* ^2^ = 0.150). However, there were no significant differences in total glutathione content (GSH + 2GSSG), GSH content or GSH/GSSG ratios among the treatment groups (Table 6).

**TABLE 6:** Superoxide dismutase activity (SOD, U/g bone marrow), catalase activity (CAT, U/g bone marrow), GSH (µM/g), GSSG (µM/g) and GSH/GSSG ratio (µM/µM) concentrations in bone marrow at six dpi.

Items^a^ ^,^ ^*^	Unit	Control	Low	Med-low	Med-high	High	SEM	ANOVA	Non-challenge vs. challenge^b^
GSH	µM/g	18.145	20.891	19.154	14.762	15.318	1.260	0.495	0.469
GSSG	µM/g	0.487	0.506	0.613	0.360	0.353	0.034	0.069	0.224
GSH + 2GSSG	µM/g	19.119	21.903	20.379	15.482	16.025	1.296	0.452	0.453
GSH/GSSG	µM/µM	5.093	5.002	5.137	4.574	4.470	0.288	0.935	0.244
CAT	U/g	32.290	30.271	26.408	27.419	44.407	3.124	0.386	0.937
SOD	U/g	4.574^bc^	4.385^c^	5.616^ab^	5.892^a^	5.434^abc^	0.188	0.027	0.488

a Low, the lowest challenge dose; Med-low, the medium-low challenge dose; Med-high, the medium-high challenge dose; High, the highest challenge dose.

b The comparisons between non-challenge control and pooled challenged groups (Low, Med-low, Med-high, and High) were calculated by unpaired *t*-test with Welch’s correction.

c GSH, glutathione content; GSSG, oxidized glutathione content; GSH + 2GSSG: the total glutathione level; GSH/GSSG, the ratio of reduced glutathione content to oxidized glutathione content; CAT, catalase activity; SOD, superoxide dismutase.

^a, ab, abc,c^ Treatments with different letters means a significantly difference between treatments by using Tukey’s HSD, test, *p < 0.05*, N = 6.

**FIGURE 3:**
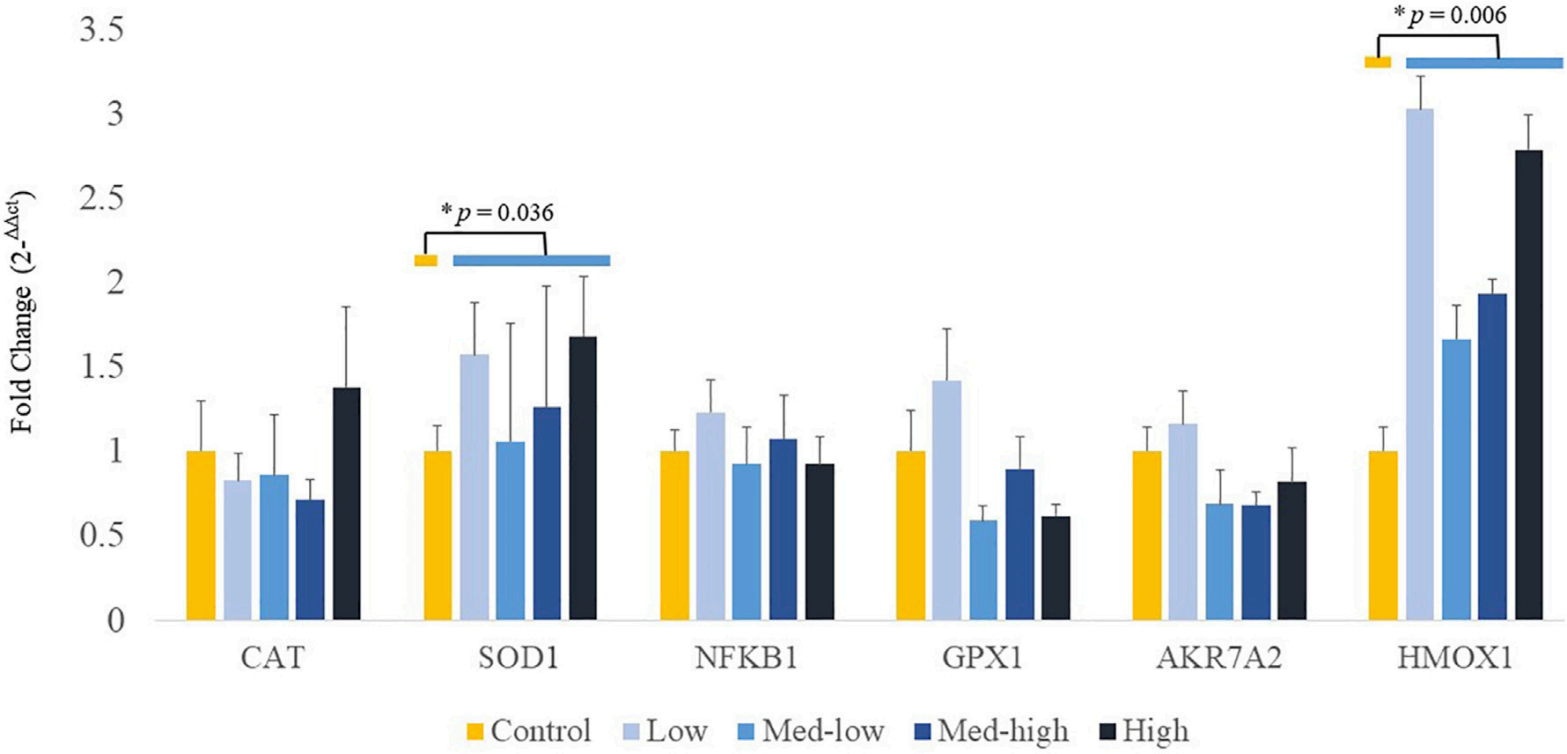
Effects of increasing oocysts dose of *Eimeria* mix on the expression of antioxidant-related transcript genes in bone marrow of broilers. Low, the lowest challenge dose; Med-low, the medium-low challenge dose; Med-high, the medium-high challenge dose; High, the highest challenge dose. CAT, catalase; SOD1, superoxide dismutase 1; NFKB1: nuclear factor kappa B subunit 1; GPX1: glutathione peroxidase 1; HMOX1: heme oxygenase 1; and AKR7A2: aflatoxin aldehyde reductase. ^a, ab, b^ Treatments with different letters means a significantly difference between treatments by using Tukey’s HSD test, *p* < 0.05, N = 6. * a significant difference between non-challenge control and pooled challenge groups (Low, Med-low, Med-high, and High) by using Welch’s *t*-test, *p <* 0.05.

Additionally, mRNA expression of *CAT* was positively correlated with higher inoculation doses of the mixed *Eimeria* oocysts in bone marrow (Figure 3; ANOVA, *p* > 0.050; linear regression, *p* = 0.040, *R* ^2^ = 0.124), whereas there were no significant differences in expression of *HMOX1*, *SOD1*, *GPX1* or *NFKB1* among the treatments in bone marrow (Figure 3; ANOVA, *p* > 0.050; linear regression, *p* > 0.050). By pooling all infected groups (Low, Med-low, Med-high, and High) together and compared with the non-infected Control, *Eimeria* infection significantly increased the mRNA level of *SOD1* (*p* = 0.036) and *HMOX1* (*p* = 0.006).

### Antioxidant Status in the Liver in Response to *Eimeria* Challenge

In the liver, CAT activity was not significantly affected by *Eimeria* infection (ANOVA, *p* > 0.050), but the activity of CAT was negatively correlated to the higher challenge dose of *Eimeria* oocysts (Table 7; linear, *p* = 0.043, *R* ^2^ = 0.143). GSH content was significantly decreased by *Eimeria* infection and the lowest GSH content was observed in the Med-High group (Table 7; ANOVA, *p* = 0.036). By comparing infected groups (Low, Med-low, Med-high, High) with the non-infected Control, a significant higher SOD activity (*p* < 0.001) and numeric lower GSH content (*p* = 0.091) were detected in pooled *Eimeria*-infected groups.

**TABLE 7:** Superoxide dismutase activity (SOD, U/g), catalase activity (CAT, U/g) and GSH content (µM/g) in the liver at six dpi.

Items^a^ ^,^ ^b^	Unit	Control	Low	Med-low	Med-High	High	SEM	ANOVA	Non-challenge vs. challenge^c^
SOD	U/g	3,891.0	16,676.5	10,079.2	13,335.7	13,097.8	60.4	0.166	<0.001
CAT	U/g	6749.8	6851.56	4,361.84	5,402.07	5,253.27	2029.01	0.778	0.594
GSH	µM**/**g	11.562^a^	7.876 ^ab^	6.710 ^ab^	6.374^b^	7.688 ^ab^	0.600	0.036	0.091

a Low, the lowest challenge dose; Med-low, the medium-low challenge dose; Med-high, the medium-high challenge dose; High, the highest challenge dose.

b GSH, glutathione content; CAT, catalase activity; and SOD, superoxide dismutase.

c The comparisons between non-challenge control and pooled challenged grops (Low, Med-low, Med-high, and High) were calculated by unpaired *t*-test with Welch’s correction.

^a,ab,b^ Treatments with different letters means a significantly difference between treatments by using Tukey’s HSD, test, *p < 0.05*, N = 6.

**FIGURE 4:**
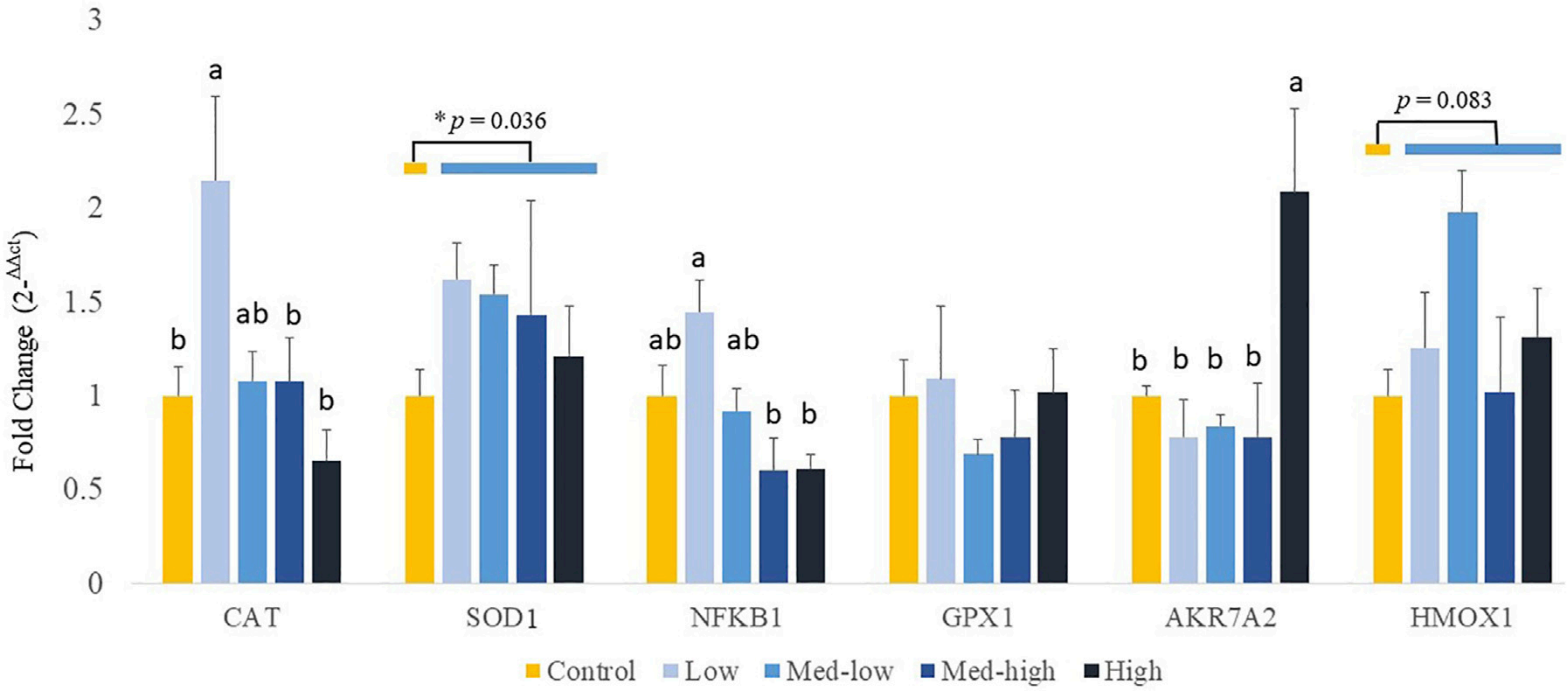
Effects of increasing oocysts dose of *Eimeria* mix on antioxidant-related transcripts gene expression in the liver of broilers. Low, the lowest challenge dose; Med-low, the medium-low challenge dose; Med-high, the medium-high challenge dose; High, the highest challenge dose. CAT, catalase; SOD1, superoxide dismutase 1; NFKB1, nuclear factor kappa B subunit 1; GPX1, glutathione peroxidase 1; HMOX1, heme oxygenase 1; and AKR7A2, aflatoxin aldehyde reductase. ^a, ab, b^ Treatments with different letters means a significantly difference between treatments by using Tukey’s HSD test, *p* < 0.05, N = 6. * a significant difference between non-challenge control and pooled challenge groups (Low, Med-low, Med-high, and High) by using Welch’s *t*-test, *p <* 0.05.

Additionally, the expression of genes coding for front-line antioxidant enzymes was measured (Figure 4). The gene expression of antioxidant gene showed a dose dependent manner. More specifically, the Low challenge dose significantly increased the mRNA expression of CAT when compared with the Control, and the expression level was decreased with the high challenge dose of *Eimeria* oocysts (Figure 4; ANOVA, *p* = 0.006; linear regression, *p* = 0.004, *R* ^2^ = 0.144). The highest inoculation dose of *Eimeria* oocysts upregulated the expression of *AKR7A2* (ANOVA, *p* = 0.006; linear regression, *p* = 0.002, *R* ^2^ = 0.434). The low challenge dose of *Eimeria* oocysts resulted in significantly higher *NFKB1* expression*,* compared to the Med-high and the High group (ANOVA, *p* = 0.002; linear regression, *p* = 0.006, *R* ^2^ = 0.263). No significant differences in the expression of *SOD1, GPX1*, and *HMOX1* were evident among the challenge doses (*p* > 0.100). By comparing infected groups with the non-infected Control, a significant higher level of *SOD1* (*p* = 0.036) and a numeric higher level of *HMOX1* (*p* = 0.083) were detected in the *Eimeria*-infected groups.

### Correlation Between Antioxidant Enzymes Level and Bone Parameters

**FIGURE 5:**
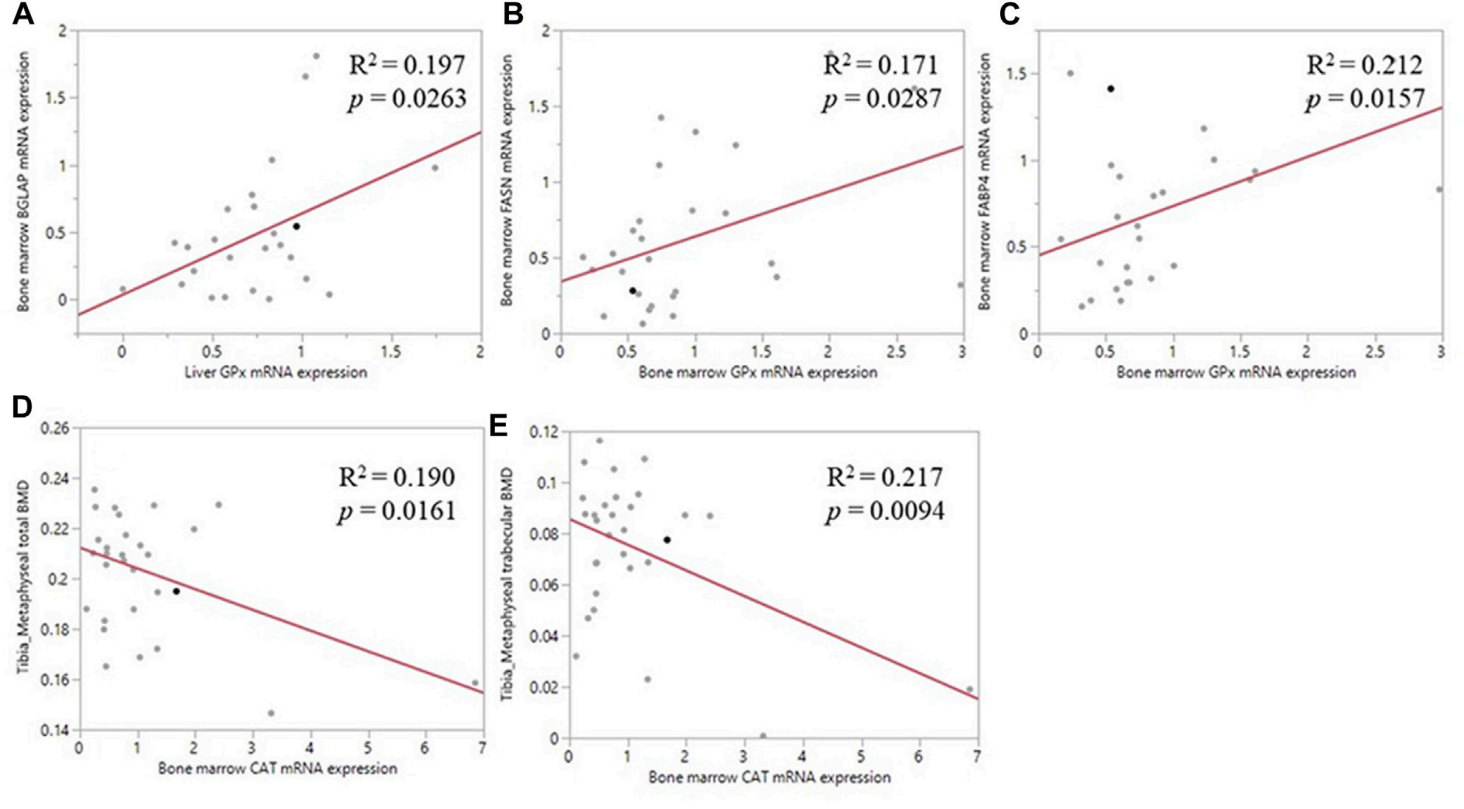
Correlation of antioxidant enzyme mRNA expression and bone related parameters. **(A)** The positive correlation between liver *GPX1* mRNA level and the bone marrow *BGLAP* mRNA expression **(B)** The positive correlation between bone marrow *GPX1* mRNA expression and the bone marrow *FASN* mRNA expression **(C)** The positive correlation between bone marrow *GPX1* mRNA expression and the between bone marrow *FABP4* mRNA expression **(D)** The negative correlation between bone marrow *CAT* mRNA expression and tibia metaphyseal total BMD **(E)** The negative correlation between bone marrow *CAT* mRNA expression and tibia metaphyseal trabecular BMD.

Pearson correlation analyses revealed a positive correlation between the liver *GPX1* mRNA level and bone marrow *BGLAP* mRNA level (*R* ^2^ = 0.197, *p* = 0.026; Figure 5A); between bone marrow *GPX1* mRNA and bone marrow *FASN* mRNA expression (*R* ^2^ = 0.171, *p* = 0.029; Figure 5B); and between bone marrow *GPX1* mRNA and bone marrow *FABP4* mRNA expression (*R* ^2^ = 0.212, *p* = 0.016; Figure 5C). Meanwhile, bone marrow *CAT* mRNA level was negatively correlated with tibia metaphyseal BMD (*R* ^2^ = 0.190, *p* = 0.016; Figure 5D) and trabecular BMD (*R* ^2^ = 0.217, *p* = 0.009; Figure 5E).

## Discussion

Based on current data, we concluded that high challenge dose of *Eimeria* infection negatively affected the long bone development. The structural changes of tibia and decreased mineral content were mainly located at the trabecular bone of metaphyseal area. The change of redox and impaired antioxidant status following the *Eimeria* infection were observed in the liver and bone marrow of broilers. Compared with the slower growing strains of broilers, bone formation and turnover are extremely rapid in the modern strains (Yair et al., 2017), that fast body weight gain places challenges to bone health in the modern broiler industry (Edwards, 2000; Fleming, 2008; Wideman, 2016; (Schmidt et al., 2009; Alrubaye et al., 2020). The rapid bone growth results in decreased mineral density, increased cortical porosity, and altered biomechanical properties of long bone in the modern broiler strains (Williams et al., 2004), that is partly responsible for broiler leg bone disorder that restricts the growth of broiler. Long bone homeostasis is closely associated intrinsic and extrinsic factors including nutrition status, physical stress (mechanical loading), immune status, hormonal status, genetics, management, and age of animals (Fleming, 2008; Rokavec and Zupan Semrov, 2020; Cao et al., 2021). Therefore, we propose that bone traits can be used as a dynamic indicator for growth and health status of poultry.

Bone is made up of two components: the organic matrix and the inorganic matrix (Rath and Durairaj, 2022). Crystals of calcium phosphate make up the bulk of the inorganic matrix, which is eventually counted as bone mineral content. The inorganic mineral content is the major component of the bone that provides stiffness and strength to the bone (Eliaz and Metoki, 2017), where quantitate bone mineral content is the easiest and most common way to reflect the bone health status. To date, researchers have conventionally focused on the changes in bone mineral content and density after *Eimeria* infection (Fetterer et al., 2013; Akbari Moghaddam Kakhki et al., 2019; Oikeh et al., 2019). Bone microarchitecture is a predictor for evaluating bone quality and health independent of bone mineral content (Brandi, 2009; Chen and Kim, 2020). However, few data exist on the poultry bone microstructure changes after *Eimeria* infection. Micro-CT is a precise and non-destructive evaluation approach that can provide a comprehensive overview of the morphological and architectural characteristics in poultry bones (Chen and Kim, 2020). In the current study, micro-CT was used in assessing the three-dimensional structure, which provides in-depth understanding behind the relationship between changes of bone traits and *Eimeria* infection. We mainly evaluated those parameters representing metaphyseal bone traits to reflect earlier bone changes under *Eimeria* spp*.* infection, because acute trabecular bone loss following infectious diseases or bone damage occurred almost exclusively within the metaphyseal compartment in human and poultry (Mubarak et al., 2009; Raehtz et al., 2018). Consistent with previous findings (Akbari Moghaddam Kakhki et al., 2019; Oikeh et al., 2019), the present results showed that a significant reduction in tibia metaphyseal bone mineral content and bone mineral density in the *Eimeria*-challenged groups, especially in the High challenge group of broilers as compared to the non-infected Control, demonstrated the impaired metaphyseal trabecular microstructure under parasite infection. The organization of the trabecular bone is not only a key to bone strength but also plays an important role in metabolic function (Seifert and Watkins, 1997). The trabecular bones are organized as a lattice structure that provides larger surface areas for osteoclast attachment, showing a higher turnover rate during bone resorption compared with cortical bones in rodent or human study (Baron et al., 1984; Qiu et al., 2019). Previous studies in mouse displayed a decreased total bone BV/TV, trabecular BV/TV, and trabecular BS, which indicated an accelerated trabecular bone turnover (Boskey and Imbert, 2017). As for both whole metaphyseal structure and trabecular bone structure in the current study, there were no statistical changes in bone mass (BV or TV) with different *Eimeria*-challenge dosages, whereas the total bone BMD and trabecular bone BMD were negatively correlated with increased inoculation doses of oocysts, and BMC decreased correspondingly. In the present study, the lower bone mineral content and density, and changed ratio of BV/TV at metaphyseal trabecular bone could be the outcome of trabecular bone remodeling in the High dose of *Eimeria* challenge group. Trabecular microstructure assay in this study also showed significant decreases in trabecular number (Tb. N) and connectivity density (Conn. Dn), and significant increases in SMI in tibia metaphyseal trabecular bone by *Eimeria* spp. challenge. The similar alteration of bone microstructures is also known in human bone microfracture, where small fractions that resulted from trauma, physical stress, or infection (Prat-Fabregat and Camacho-Carrasco, 2016; Solomon et al., 2018). Bone trabecular microstructure traits such as SMI was designed to estimate the rod or plate-like trabecular geometry which describes the trabecular network (Hildebrand and Ruegsegger, 1997). Evaluation of SMI across human and rat studies suggests that higher SMI values represent a rod-like trabecular structure that indicates a poor weight-bearing ability; this structure could be observed in osteoporosis disease models (Borah et al., 2004; Akhter et al., 2007). Higher SMI values and lower numbers of trabeculae indicated a poor trabecular bone architecture in human (Greenwood et al., 2015). As for the current study, a higher SMI and lower number of trabeculae pointed out the poor-quality of the trabecular bone in *Eimeria* challenge groups, indicating that the bone mineral loss might have happened before any observation of phenotype abnormality of tibia bone during the *Eimeria* infection. At 19 days of age, the body weight of the broiler has yet to cause mechanical trauma on the tibia, thereby the immune response, nutrient deficiency or immune response associated energy cost resulted in the long bone structure abnormality and bone mineral loss. Although changes in bone microstructure during *Eimeria* infection may not necessarily cause bone damage during growth, it potentially enhances the risk of bone damage and the susceptibility to bone disease, with certain mechanical triggering and severe intestinal bacterial infection (Prisby et al., 2014; Wideman, 2016). Moreover, in the current study, the mRNA expression of bone related proteins in bone marrow is also in line with the micro-CT morphological observation. A reciprocal relationship between a key osteogenic marker, *BGLAP,* and a key adipogenic marker, *PPARG* was observed in the Med-high group that were challenged with second highest dose of *Eimeria* oocytes. Bone marrow adipose tissue content has adverse effects on bone quality and can serve as a relevant marker of a compromised bone integrity (Hardouin et al., 2016; Sundh et al., 2016; Kim et al., 2017). Previous studies indicated that higher *PPARG* expression could direct the mesenchymal stem cells (MSCs) differentiated into adipocytes instead of osteoblasts *in vitro* (Shockley et al., 2009; Hu et al., 2018). In the present study, the suppressed expression of *BGLAP* and increased expression of *PPARG* both indicated the impetus of fat growth instead of bone formation during *Eimeria* infection, confirming the negative impact of *Eimeria* infection on bone health from mRNA level. However, by *Eimeria* infection and intestinal damage, the dietary lipid malabsorption and low nutrient levels in high challenge dosage groups might predispose the suppression of fatty acid synthesis by decreasing the expression of *FASN* in bone marrow.

Broiler bone majorly develops in the first 3 weeks of life (Lilburn et al., 1994; Williams et al., 2000), which overlaps with the timeline when the oocyst shedding rapid accumulated (Chapman et al., 2014). For the etiology of bone loss after *Eimeria* infection, other than the main factors such as physical stress or nutritional deficiency, accumulating data documented the interaction between immune response, oxidative stress and bone mineral loss due to the crosstalk between the skeletal and the immune systems, and the important biological role of ROS in a variety of physiological systems (Takayanagi, 2007; Lorenzo et al., 2008). Reactive species play an important role in immune response. As for innate immunity, immune cells such as macrophages and neutrophils (heterophils in avian species) utilized phagocytic oxidative burst to destruct pathogens (Lauridsen, 2019; Mishra and Jha, 2019). However, unregulated ROS can damage host tissue homeostasis (Costantini and Moller 2009; Rehman et al., 2018). Coccidiosis can cause severe oxidative stress that elevates intracellular levels of reactive oxygen species (ROS) in broiler and other species (Sepp et al., 2012; Abdel-Haleem et al., 2017). *In vitro* studies in human and mouse cells have shown that ROS is an important activator for various cell signaling pathways, mediated MSCs differentiation and cell fate (Yang et al., 2013; Atashi et al., 2015). Accumulating evidence suggesting the alteration of the redox state causes systemic changes that can coordinate osteoblast differentiation or osteoclast activity that relat to the bone remodeling process in human and animal models (Domazetovic et al., 2017; Schreurs et al., 2020; Sheppard et al., 2022). The supplementation of trace minerals can alleviate the negative effect of oxidative stress and optimize the bone quality in human and broilers (Santos et al., 2020; Savaram Venkata et al., 2021). Infection with *E. tenella* or *E. acervulina* could increase serum CAT activity while it decreases serum GPX activity in broilers (Georgieva et al., 2006). In the present study, the changes in antioxidant enzyme activity and level of mRNA expression in the liver and bone marrow displayed a systemic oxidative stress in broilers after *Eimeria* infection. Moreover, significantly lower GSH levels were observed in the liver of *Eimeria* infected birds. The decreased defensive antioxidant abilities in higher *Eimeria* challenge dosage groups could partially be related to reduced intestinal absorption of antioxidants (Georgieva et al., 2006; Abdel-Haleem et al., 2017; Mishra and Jha, 2019). Moreover, in the present study, the mRNA expression pf *HMOX1* was upregulated by *Eimeria* infection. HMOX plays essential roles against oxidative stress by balancing body’s systemic iron homeostasis and inflammation response (Otterbein and Choi, 2000; Immenschuh et al., 2010). *In vitro* studies have shown that upregulated *HMOX*1 inhibited the maturation and mineralization of osteoblasts (Lin et al., 2010), and HMOX enzyme was involved in the response of bone marrow macrophages to RANKL, which is an essential pathway for osteoclast formation (Florczyk-Soluch et al., 2018). Thus, the increased mRNA expression of *HMOX1* in bone marrow may be associated with oxidative- or inflammation-induced bone loss by either increasing the activity of osteoclast formation, inhibiting maturation of osteoblast, or both.

Unlike previous results that enzymatic antioxidant SOD was remarkably decreased in chicken serum in most cases of *Eimeria* spp. infection (Georgieva et al., 2006), we found the enzyme activity of SOD and mRNA expression of *SOD1* were significantly increased in the liver and bone marrow. Previous researchers have established that SOD has immunomodulatory function, and SOD3 is reported to downregulate several signaling cascades including nuclear factor kappa B (NFKB) transcription factors, thereby constraining the inflammatory responses in MSCs (Sah et al., 2020). In the present study, significantly downregulated mRNA expression of NFKB1 was coupled with upregulated mRNA expression of *SOD1* in the liver, indicating the constraining of inflammatory responses. SODs also play significant role in MSCs differentiation and function (Nightingale et al., 2012; Shi et al., 2019). An *in vitro* study of human MSCs reported that the expression of *SOD3* was significantly increased under adipogenic differentiation, and overexpression of *SOD3* in MSCs promoted adipogenic differentiation of MSCs *in vitro* instead of osteogenic differentiation (Nightingale et al., 2012). Therefore, the increased SOD enzyme activity, increased mRNA expression of *SOD1*, and increased expression of *PPARG* both in the present study indicated that oxidative stress caused by *Eimeria* infection tilts the balance of MSC lineage specific differentiation in bone marrow more toward the adipogeneic differentiation.

Another crucial enzyme antioxidant, catalase (CAT), has been described as an important enzyme implicated in inflammation conditions (Georgieva et al., 2006). In present study, mRNA expression and activity of *CAT* were negatively correlated with higher challenge dose of *Eimeria* spp. in the liver. The Low group showed a higher level of *CAT* mRNA expression when compared with the Control, but higher *Eimeria* infection dosage did not change the expression of *CAT* in the liver. In bone marrow, mRNA expression of *CAT* was positively correlated with higher challenge dose of *Eimeria* spp. in bone marrow, where this result is similar with other studies that *Eimeria* infection decreased serum GPX activity but increased serum CAT activity (Georgieva et al., 2006; Georgieva et al., 2011). However, the enzyme activity of CAT in bone marrow was not affected by *Eimeria* spp*.* infection at six dpi, suggested that bone marrow is not a CAT active site during *Eimeria* infection, but lower level of *Eimeria* infection can stimulate synthesis of enzymatic antioxidant in bone marrow (Zhang et al., 2019). The high dose of inoculation might lead to apoptotic cell death that negatively impacts on protein level and mRNA level of CAT (Saha et al., 2020). Furthermore, the correlation analysis indicated that liver *GPX1* mRNA expression is positively correlated with bone marrow *BGLAP* mRNA expression, whereas bone marrow *CAT* mRNA expression was negatively correlated with tibia metaphysis BMD, emphasizing that the negative impact of *Eimeria* infection on bone quality might be associated with the occurrence of oxidative stress. However, while nutritional factors and animal husbandry play significant roles in determining antioxidant status and bone homeostasis**,** *in vitro* animal model is hard to provide direct evidence to explain the interaction between oxidative stress and bone remodeling in broilers. Further studies require comprehensive *in vitro* and in *ovo* investigation, which is necessary to confirm the functional significance of antioxidants in bone homeostasis, especially under parasite challenge models.

Recent studies also indicated that the immune status of individuals promoted the process of osteoclastic bone resorption that resulted in bone mineral loss, which is defined as osteoimmunology (Kamibayashi et al., 1995; Solomon et al., 2018). For example, in human clinical studies, the long-term investigation showed bone microarchitectural changes under infection of C virus (HCV), which increased the risk of fracture (Bedimo et al., 2018). Acute malaria infection severely suppresses bone homeostasis, which leads to increased RANKL expression and overstimulation of osteoclastogenesis which favors bone resorption (Lee et al., 2017). Trabecular bone microstructure is impaired in the proximal femur of human immunodeficiency virus-infected (HIV) men with normal bone mineral density (Kazakia et al., 2018). Decreased bone mass and abnormality in trabecular and cortical microarchitecture were observed in young men infected with HIV early in life (Yin et al., 2014). Related reports are very limited in poultry studies. Studies of acute inflammatory response caused by lipopolysaccharides (LPS) injection suppressed growth performance and altered bone homeostasis, which significantly decreased body weight and tibia breaking strength (Mireles et al., 2005). Immunosuppressive doses of dexamethasone triggered high incidences of turkey osteomyelitis complex in turkey poults and bone lesions in broilers (Wideman and Pevzner, 2012). All those studies indicated the link between health status and bone remodeling in broilers, suggesting immune status must be considered as another critical factor in the pathogenesis of bone abnormities under intestinal parasite infection.

## Data Availability

The original contributions presented in the study are included in the article/Supplementary Materials, further inquiries can be directed to the corresponding author.
